# Lamin B1 as a key modulator of the developing and aging brain

**DOI:** 10.3389/fncel.2023.1263310

**Published:** 2023-08-31

**Authors:** Foteini-Dionysia Koufi, Irene Neri, Giulia Ramazzotti, Isabella Rusciano, Sara Mongiorgi, Maria Vittoria Marvi, Antonietta Fazio, Minkyung Shin, Yoichi Kosodo, Ilaria Cani, Elisa Giorgio, Pietro Cortelli, Lucia Manzoli, Stefano Ratti

**Affiliations:** ^1^Cellular Signalling Laboratory, Department of Biomedical and Neuromotor Sciences (DIBINEM), Anatomy Centre, University of Bologna, Bologna, Italy; ^2^Korea Brain Research Institute (KBRI), Daegu, Republic of Korea; ^3^Department of Biomedical and Neuromotor Sciences (DIBINEM), University of Bologna, Bologna, Italy; ^4^Department of Molecular Medicine, University of Pavia, Pavia, Italy; ^5^Medical Genetics Unit, IRCCS Mondino Foundation, Pavia, Italy; ^6^IRCCS Istituto Delle Scienze Neurologiche di Bologna, Bologna, Italy

**Keywords:** lamin B1, nuclear lamina, glia, astrocytes, neurogenesis, neurons, brain development, aging brain

## Abstract

Lamin B1 is an essential protein of the nuclear lamina that plays a crucial role in nuclear function and organization. It has been demonstrated that lamin B1 is essential for organogenesis and particularly brain development. The important role of lamin B1 in physiological brain development and aging has only recently been at the epicenter of attention and is yet to be fully elucidated. Regarding the development of brain, glial cells that have long been considered as supporting cells to neurons have overturned this representation and current findings have displayed their active roles in neurogenesis and cerebral development. Although lamin B1 has increased levels during the differentiation of the brain cells, during aging these levels drop leading to senescent phenotypes and inciting neurodegenerative disorders such as Alzheimer’s and Parkinson’s disease. On the other hand, overexpression of lamin B1 leads to the adult-onset neurodegenerative disease known as Autosomal Dominant Leukodystrophy. This review aims at highlighting the importance of balancing lamin B1 levels in glial cells and neurons from brain development to aging.

## Graphical Abstract

Summary of the involvement of lamin B1 in the different processes of brain development and aging. Created with BioRender.com. 
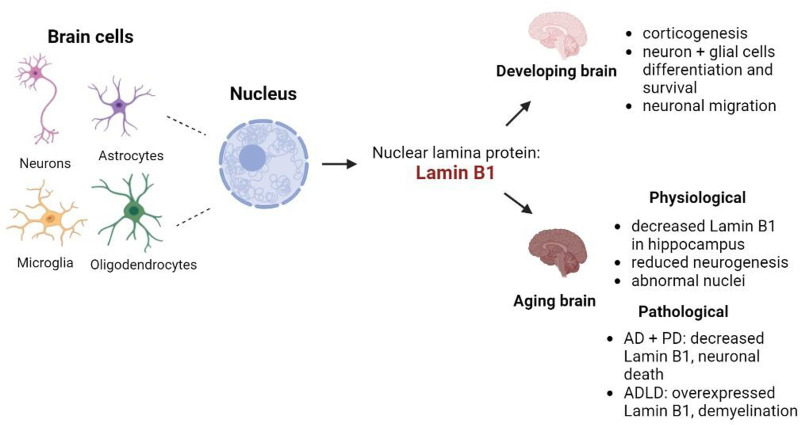

## 1. Introduction

Nuclear lamins are type V intermediate proteins forming a filamentous high-ordered meshwork near the inner nuclear membrane known as nuclear lamina (Burke and Stewart, 2013; Tenga and Medalia, 2020; Murray-Nerger and Cristea, 2021; Evangelisti et al., 2022). The nuclear lamina is an evolutionary conserved feature of all metazoan nuclear envelopes and nuclear lamins are thought to be the most ancient intermediate filaments (Xie et al., 2016; Murray-Nerger and Cristea, 2021). Lamins in mammals, depending on their sequence homologies, are subdivided into four categories: lamins A, C, B1, and B2 (Jung et al., 2013a; Evangelisti et al., 2022). Like mammals, *Drosophila* and zebrafish possess A-type and B-type lamins of great similarity to the evolutionary conserved human lamins, whereas the *C. elegans* genome contains a single B-type lamin gene (Ben-Harush et al., 2009; Verma and Parnaik, 2015; Tenga and Medalia, 2020; Bitman-Lotan and Orian, 2021; Lin et al., 2022; Morgunova et al., 2022).

**FIGURE 1:**
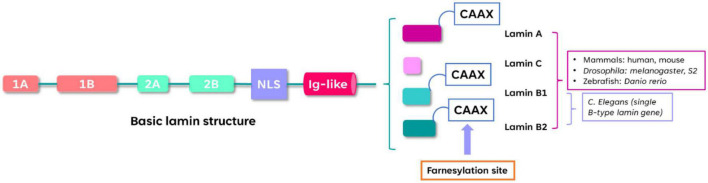
Structure of lamins. The α-helical rod domain contains Coils 1A, 1B, 2A, 2B separated by linker segments. The C-terminal tail contains the nuclear localization signal (NLS), immunoglobulin-like domain (Ig-like) and the conserved CAAX motif that undergoes farnesylation. The lamin-protein domains organization is conserved among different species such as mammals, zebrafish, *Drosophila* and *C. elegans.*

All lamins share a set of domains: head, coiled-coil rod, and globular Ig-like fold (Tenga and Medalia, 2020; Murray-Nerger and Cristea, 2021). The splice-variant derived A-type lamins include the two-major (lamin A and lamin C) and the two minor (lamin C2 and lamin AΔ10) isoforms and are encoded by a single gene in humans (*LMNA* on chromosome 1q11-q2). Lamin A is translated as the precursor protein prelamin A that undergoes post-translational modification leading to mature lamin A (Burke and Stewart, 2013; Chiarini et al., 2019; Murray-Nerger and Cristea, 2021; Evangelisti et al., 2022). The two major mammalian B-type lamins (lamin B1 and lamin B2) are, respectively encoded by the genes *LMNB1* (5q23.2-q31.3) and *LMNB2* (19p13.3) in humans, and are widely expressed in somatic cells (Burke and Stewart, 2013; Evangelisti et al., 2022). Like lamin A, lamin B1, and lamin B2 contain a CAAX termination motif that undergoes extensive post-translational modifications (farnesylation) (Jung et al., 2013a). It has been demonstrated that farnesylation of Lamin B1, but not Lamin B2, is crucial for brain development (Jung et al., 2013b). The structure of lamins is presented in Figure 1.

The classification of A- and B-type lamins is based on their different biochemical properties (Jung et al., 2013a). Their functions include defining nuclear shape, providing mechanical stability and structural support, participating in stress responses, regulating chromatin organization and gene expression, affecting DNA replication and repair, and contributing to cell cycle progression (Murray-Nerger and Cristea, 2021; Evangelisti et al., 2022). A-type lamins can freely diffuse in the nucleoplasm, whereas B-type lamins are mainly localized at the nuclear membrane most likely due to their permanently farnesylated state (Evangelisti et al., 2022). The ratio of A- to B-type lamins is associated with the contribution of each type to nuclear stiffness. Particularly, recent studies have shown that lamin A mostly contributes to viscosity providing an anti-rupture effect, while B-type lamins and lamin A/C contribute to stiffness and nuclear deformation (Wintner et al., 2020; Murray-Nerger and Cristea, 2021).

Moreover, there is a different ratio of A- to B-type lamin expression during the different developmental stages. A-type lamin expression (lamins A and C) starts during the later stages of embryonic development with a postnatal peak, whereas B-type lamins (lamins B1 and B2) are expressed from the earliest developmental stages in all cell types (Dechat et al., 2010; Jung et al., 2013a). In particular, A-type lamins are associated with differentiated mesenchymal cells characterized by stiffer cells and nuclei whereas neurons and glia in the central nervous system (CNS) have high levels of lamin C expression and infinitesimal expression of lamin A. On the contrary, B-type lamins are expressed in all cell types and are necessary for physiological organogenesis including neuronal migration and patterning during brain development (Vergnes et al., 2004; Lammerding et al., 2006; Coffinier et al., 2011; Jung et al., 2012; Nmezi et al., 2019).

The focus of this review is to elucidate the key role of lamin B, particularly lamin B1, in brain development and aging. As lamin B1 has been less studied with respect to other lamins, recent studies have demonstrated that lamin B1 plays a crucial role in neurogenesis and cerebral development. This review aims at summarizing recent findings on the effects of the different levels of lamin B1 in glial cells and neurons from the development to the aging of the brain.

## 2. Roles of different brain cells in brain development

Human brain development is characterized as a complex series of dynamic processes that take place throughout the developmental stages with the goal of promoting the emergence and differentiation of new neural structures and functions. It begins in the third gestational week (GW) with the differentiation of the neural progenitor cells, extending to late adolescence and throughout the lifespan (Stiles, 2008; Stiles and Jernigan, 2010). By the end of the embryonic period (GW8), brain structures and CNS are defined and during the fetal development period (until the end of gestation) a rapid growth and elaboration of cortical and subcortical structures is observed (Kostović and Jovanov-Milosević, 2006; Stiles and Jernigan, 2010).

The process of brain development is highly dependent on the generation of the brain cells. Neurons and glia are the two major brain cell types, and their generation is a consequence of an exceptionally organized sequence of events (Stiles and Jernigan, 2010; Budday et al., 2015). In the following paragraphs, neurodevelopment, and the roles of glia in the brain are reported.

### 2.1. Neurogenesis and neuron migration

Neurogenesis begins with the formation of the neocortex at the rostral end of the neural tube, near the outer surface of the embryonic cerebral vesicle, with the closing of the neural tube at embryonic day 30 (GW5) in humans (Sidman and Rakic, 1973; O’Rahilly and Müller, 2005; Budday et al., 2015). The onset of neurodevelopment includes cell division and cell migration that result in the formation of the six layered cortex (Budday et al., 2015). Neuronal progenitors are post-mitotic cells that divide to form new cells. From the end of gastrulation to embryonic day 42 (E42), the progenitors perform “symmetrical” division meaning that two identical neural progenitor cells are produced. Cortical neurogenesis in humans is an ongoing process until around E108 (Clancy et al., 2001; Wodarz and Huttner, 2003; Huttner and Kosodo, 2005; Stiles and Jernigan, 2010).

Most neurons are produced in the ventricular zone (VZ) located at the center of the brain and migrate radially out to the developing neocortex. In the early stages of neocortical development, the neurons travel small distances and use a migration mode known as somal translocation (Nadarajah et al., 2003; Stiles and Jernigan, 2010). As the development proceeds, the brain becomes larger, and the neurons must cover longer distances. Therefore, the migration is now supported by a special population of cells within the VZ called “radial glial guides” (Rakic, 1972; Huttner and Kosodo, 2005; Kosodo and Huttner, 2009). When neuronal migration is almost completed, cortical neurons begin to connect with other neurons. Neuronal connectivity involves the growth of neuronal dendrites and axons, the formation of synapses, the development of the vasculature system and the generation and expansion of glial cells (Budday et al., 2015).

### 2.2. Formation of glial cells and their roles in brain development and function

During the postnatal period, neurodevelopment is more limited, but new neurons still emerge in the subventricular zone (SVZ) and migrate to the olfactory bulb. In addition, neurons are produced in the hippocampal dentate gyrus (DG) and migrate from the subgranular layer to the nearby granular layer. This type of neurogenesis continues throughout adulthood with only a small amount of neuronal population produced. Contrastingly to neurogenesis, proliferation and migration of glial progenitors continues throughout adult life (Cayre et al., 2009; Stiles and Jernigan, 2010).

Glial progenitors (particularly oligodendrocyte progenitor cells, OPC) continue appearing in the adult brain in a wide anatomical distribution and can differentiate in response to injury. Their proliferation takes place at the forebrain subventricular zone, and they migrate outward into the overlying white matter and cortex, striatum, and hippocampus, where they differentiate into oligodendrocytes and astrocytes (Cayre et al., 2009; Stiles and Jernigan, 2010). The three types of glial cells in the CNS are oligodendrocytes, astrocytes and microglia and they constitute 90% of the human brain cells. Notably, recent studies have overturned their representation as supportive cells to neurons and have demonstrated their essential roles in brain development and function (Haydon, 2001; Doetsch, 2003; Allen and Barres, 2005; Seifert et al., 2006; He and Sun, 2007).

Glial cells contribute to the formation of the blood-brain barrier, the impermeable lining of the brain capillaries and venules, that prevents toxic substances in the blood from entering the brain. Regarding the specific contributions of the different glial cells, oligodendrocytes support myelination by wrapping themselves around axons to form myelin sheaths which are the most susceptible structures to brain damage (McLaurin and Yong, 1995; Diemel et al., 1998; Simons and Trajkovic, 2006; Barres, 2008). Interestingly, myelination in humans mainly occurs postnatally when neuronal migration has ended and lasts for decades, whereas in rodents, myelination starts from neurogenesis and takes only a few weeks. Even though oligodendrocytes in humans are more and larger than in rodents, their density per white matter volume in humans and rodents is very similar (Bradl and Lassmann, 2010; Budday et al., 2015). Another type of glial cells are astrocytes that are the most abundant brain cell type with human astrocytes being larger and more complex and diverse than the rodent ones (Nedergaard et al., 2003; Budday et al., 2015). Astrocytes facilitate rapid removal of synaptic glutamate after its release from the presynaptic terminal preventing glutamate-mediated neurotoxicity. Lastly, unlike oligodendrocytes and astrocytes, microglia are the smallest glial cells that act as phagocytes of the CNS and are recruited upon infection and injury (Morrow et al., 2001; He and Sun, 2007; Freeman, 2010; Molofsky et al., 2012; Budday et al., 2015). Microglia arise from macrophages outside the nervous system contributing to the proliferation and differentiation of neurons, clearing debris and remodeling synapses (He and Sun, 2007; Harry, 2013; Yang et al., 2013; Budday et al., 2015).

## 3. The role of lamin B1 in the developing brain

It is widely recognized that lamin B1 plays an important role in general development. It has been reported that mice with a mutant form of *Lmnb1* displayed developmental defects in lung and bone, and the fibroblasts of embryos carrying the mutation exhibited impaired adipocyte differentiation and an increased polyploidy occurrence (Vergnes et al., 2004). Regarding brain development, over the years lamin B1 has been studied together with other lamins (A/C, and B2) for elucidating their different roles in the developmental brain processes. In this part of the review, the contribution of lamin B1 to the developing brain is summarized.

### 3.1. Lamin B1 is essential for monitoring the differentiation process of the brain cells

Starting from neurons, Takamori et al. (2007) demonstrated that the composition of nuclear lamin differs during neuronal differentiation in the two neurogenic regions (subgranular zone of DG, SGZ, and subventricular zone of the lateral ventricle, SVZ) of the adult rat brain. Particularly, three cell types were analyzed with immunostaining: glial fibrillary acidic protein (GFAP)-positive cells as primary progenitor (stem) cells, polysialylated neural cell adhesion molecule (PSA-NCAM)-positive cells as subsequent neuronal progenitor cells, and NeuN-positive mature neurons. GFAP-positive cells possessed positive levels of lamin A/C, B1 and B2, PSA-NCAM-positive cells were negative for lamin A/C, highly positive for B1 and weakly positive for B2, and mature neurons were positive for lamin A/C, weakly positive for B1 and highly positive for B2, in both neurogenic regions. Focusing on lamin B1, primary progenitor cells (GFAP-positive) have moderate levels of lamin B1 that become high when the cells become progenitors (PSA-NCAM-positive) and decrease at their late stages as progenitors and during neuronal maturation (NeuN-positive).

In another study of Takamori et al. (2018) the composition of lamin subtypes in neurons, astrocytes, oligodendrocytes and microglia in the adult rat cerebral cortex were studied. Mature neurons were examined and were negative for lamin A, highly positive for lamin C and B2, and positive for lamin B1 (Takamori et al., 2014). Neural-glial antigen 2 (NG2)-positive oligodendrocytes showed intense lamin B1 immunoreactivity for the progenitors compared to the mature oligodendrocyte cells (Dawson et al., 2000; Horner et al., 2002; Nishiyama et al., 2002; Takamori et al., 2018). Moreover, OPC (Olig2-positive) and glutathione-S-transferase-pi (GST-pi)-positive and Olig2-positive mature oligodendrocytes were both negative for lamin A and positive for lamin B2. In addition, only mature oligodendrocytes were lamin C positive. Interestingly, high lamin B1 levels were only found in OPCs. Regarding the other glial cells, astrocytes (GFAP-positive) and microglia [ionized calcium-binding adaptor molecule-1 (Iba-1)-positive], like oligodendrocytes, were negative for lamin A. Moreover, both cell types were positive for Lamin B1 and B2 with B2 being weakly positive compared to that of neurons. Regarding lamin C, microglia were negative, but astrocytes were positive with a lower intensity than that of neurons (Takamori et al., 2018).

To sum up, B-type lamins are the only ones observed in all brain cell types. Interestingly, decreasing levels of lamin B1 were observed from differentiation to maturation phase in both neurons and oligodendrocytes. Increased levels of lamin B1 could be indicative of its essential role during the differentiation stage that is a delicate cellular process, and the decreasing pattern could be signaling the upcoming stability of maturation (Stiles, 2008; Takamori et al., 2018).

### 3.2. Impact of lamin B1 post-translational modification in the developing brain

The different roles of lamin B1 and B2 were also demonstrated by Jung et al. (2013b), Lee et al. (2014a) but at a post-translational protein level involving the difference in farnesylation of lamin B1 and lamin B2. Knock-in mice that produced non-farnesylated versions of lamin B1 and lamin B2 were generated by replacing the cysteine of the CAAX motif with a serine. Knock-in mice with non-farnesylated lamin B2 were completely healthy without any detectable neuropathological or nuclear shape abnormalities in the cerebral cortex. In contrast, knock-in mice with non-farnesylated lamin B1 died soon after birth and had neuronal migration defects in the cerebral cortex.

Particularly, midbrain neurons of the non-farnesylated lamin B1 knock-in mice had dumbbell-shaped nuclei, with the nuclear lamina at one end of the dumbbell and the bulk of chromosomal DNA at the other. Dumbbell-shaped nuclei were also found in cultured neurons upon migration from neurospheres. The dumbbell-shape was justified as a consequence of defective lamin B1 anchoring to the inner nuclear lamina (Jung et al., 2013b; Lee et al., 2014a).

From the above it is evident that farnesylation of lamin B1 (and not lamin B2) is crucial for neuronal migration and chromatin retention. Thus, lamin B1 has a distinctly different role than lamin B2 in normal brain development (Jung et al., 2013b).

### 3.3. Lamin B1 deficiency and brain development

Coffinier et al. (2010, 2011), Young et al. (2012) depicted the role of lamin B1 in brain development with knock-out mice deficient of lamin B1 and lamin B2. It was reported that *Lmnb1*^Δ^ ^/Δ^ embryos have severe defects in neuronal migration (Kim et al., 2011), defective neuronal survival, and abnormal layering of neurons in the brain. Bromodeoxyuridine birth-dating experiments demonstrated that neurons born at E13.5 in wild-type mice were found in cortical plate V, whereas for *Lmnb1*^Δ^ ^/Δ^ embryos neurons were scattered throughout the cortical plate. Moreover, at E15.5 and E17.5, the cortical plate in *Lmnb1*^Δ^ ^/Δ^ embryos was abnormally thin and immunohistochemical analyses of E16.5 *Lmnb1*^Δ^ ^/Δ^ embryos revealed that neurons were in aberrant locations.

Furthermore, it was reported that the neuropathology in *Lmnb1*^Δ^ ^/Δ^ embryos was more severe than in *Lmnb2*^–/–^ embryos (Coffinier et al., 2011; Young et al., 2012). *Lmnb1*^Δ^ ^/Δ^ mice were smaller than the *Lmnb2*^–/–^ ones, and both died shortly after birth. Also, *Lmnb1*^Δ^ ^/Δ^ had nuclear shape abnormalities characterized by a large nuclear bleb. A defective layering of neurons in the brain was observed in both mice with an overall greater reduction of cortical cellularity in *Lmnb1*^Δ^ ^/Δ^ embryos. B-galactosidase staining revealed that *Lmnb1* is expressed at much higher levels than *Lmnb2* throughout the cortex and therefore this could be a possible explanation for the more severe neuropathology associated with lamin B1 deficiency. However, this statement is not enough for fully understanding whether lamin B1 and B2 have distinct roles in the developing brain.

The previous matter was addressed in studies on reciprocal knock-in mice (Lee et al., 2014a,b) that displayed increased lamin B2 production did not prevent the neurodevelopmental abnormalities related to *Lmnb1* deficiency and increased lamin B1 levels were also unable to prevent the abnormalities associated with *Lmnb2* deficiency. However, it was found that increased production of one B-type lamin can partially improve the phenotypes related to the loss of the other. Particularly, *Lmnb1*^*B*2/*B*2^ mice expressed around 3-fold more lamin B2 than *Lmnb1*^–/–^ mice and their developmental abnormalities were less severe than those of *Lmnb1*^–/–^ mice.

As a continuation of the above studies, the nuclear membrane (NM) ruptures and cell death caused by lamin B1 and B2 deficiencies in migrating neurons were investigated (Chen et al., 2019). Migrating neurons within the cortical plate of E18.5 *Lmnb1*-deficient embryos exhibited NM ruptures followed by DNA damage and cell death. Notably, non-migrating cells did not have NM ruptures within the ventricular zone. Furthermore, cultured neuron studies depicted the limited capacity of lamin B2 to substitute for lamin B1. Overexpression of lamin B2 in *Lmnb1* knock-out mice reduced but did not prevent NM ruptures and cell death, even though lamin B2 covered the entire nuclear rim. In contrast, lamin B2 overexpression in *Lmnb2* knock-out mice abolished NM ruptures. Concluding, it was suggested that lamin B1 is more important for maintaining the structural integrity of the nuclear envelope, whereas lamin B2 is crucial for NM repair.

### 3.4. Balanced lamin B1 levels are essential for corticogenesis

It is appropriate to close the part of brain development with the importance of balancing lamin B1 levels in the developing brain. It has been reported that on one hand, *LMNB1* overexpression selectively decreases axonal outgrowth, and on the other *Lmnb1* deficiency strongly impairs dendrite length and complexity (Giacomini et al., 2016).

Giacomini et al. (2016) analyzed axonal growth and dendrite development for understanding the effects of lamin B1 levels in neuronal morphogenesis. The length and the complexity of dendritic trees were not affected by *LMNB1* overexpression. In *Lmnb1*^Δ^ ^/Δ^ neurons, the axonal length was reduced at late (7 days *in vitro*, DIV) but not early (3 DIV) differentiation stages, instead dendrite development was strongly impaired in *Lmnb1*^Δ^ ^/Δ^ at all stages of differentiation. It was also found that lamin B1 is essential for proper regulation of depolarizing stimuli. Dendrite elongation and arborization is enhanced by potassium chloride (KCl) exposure, and extracellular signal-regulated kinases (ERK) signaling is required for sustaining dendrite outgrowth in both wild-type and KCl-stimulated *Lmnb1*^+/+^ neurons. However, *Lmnb1*-deficient neurons exhibit reduced nuclear ERK import and do not respond to KCl stimulus. Thus, it is evident that ERK nuclear signaling probably mediates the effects of lamin B1 on dendritic outgrowth.

As a continuation of this study, Mahajani et al. (2017) demonstrated that finely tuned levels of lamin B1 are required for neurogenesis, proper astrocyte development and corticogenesis. Particularly, it was reported that lamin B1 levels modulate the differentiation of murine neural stem cells (NSC) into neurons and astroglial-like cells with human *LMNB1* overexpression increasing the neuron proportions. Interestingly, a recent study on DYT1 dystonia human motor neurons with upregulated *LMNB1* revealed similar results with defective neuron development and abnormal nuclei with thicker nuclear lamina (Ding et al., 2021). Furthermore, Mahajani et al. (2017) displayed that *Lmnb1* depletion favored *in vitro* NSC differentiation into GFAP-immunoreactive cells over neurons without affecting oligodendrocyte generation. *In vivo Lmnb1* silencing in mouse embryonic brain led to aberrant cortical positioning of neurons and increased expression of astrocytic marker GFAP in the cortex of 7-day old pups. This indicated that *Lmnb1* silencing could act with non-cell autonomous mechanisms turning on GFAP expression, inducing gliosis and/or proliferation of resident astrocytes nearby *Lmnb1*-silenced cells. Moreover, experiments on *Lmnb1*-null embryos demonstrated the role of *Lmnb1* in corticogenesis and neuronal differentiation and migration. In *Lmnb1*-null embryos, nuclear size and neurogenesis were reduced at E13.5 and GFAP expression increased from E17.5. Regarding neuronal migration, when *Lmnb1* was silenced, cortical migration of differentiated cells was hindered. Overall, it was suggested that aberrant differentiation of neurons and premature expression of astrocytic markers could add up to the complex phenotype of *Lmnb1*-deficient developing brain.

Concluding, from all these findings it is evident that unbalanced lamin B1 has detrimental effects on neural development and maturation (Mahajani et al., 2017). Therefore, balanced lamin B1 is an indispensable prerequisite for physiological neurodevelopment and corticogenesis. A future direction of these studies could be toward the development of cerebral organoids from human induced pluripotent stem cells with overexpression and depletion of lamin B1 for studying their effects in human corticogenesis. In the table below (Table 1), a summary of the contribution of lamin B1 to the developing brain is depicted.

**TABLE 1:** Summary of lamin B1 contribution to the developing brain. In the first part of the table the lamin levels are indicated as follows: + + + ; highly positive, + + ; positive, + ; weakly positive.

Process	Model	Effects								References
Differentiation	Adult rat	**Lamin**	**NPC*^a^***	**Mature neurons**	**OPC*^b^***	**Oligodendrocytes**		**Astrocytes**	**Microglia**	Takamori et al., 2007, 2014, 2018
		**B1**	+ + +	+	+ + +	+ +		+ +	+ +	
		**B2**	+	+ ++	+	+		+	+	
Post-translational modification	Knock-in mouse	**Non-farnesylated Lamin B1**				**Non-farnesylated Lamin B2**				Jung et al., 2013b
		Death shortly after birth, neuronal migration defects, dumbbell shaped nuclei				Healthy				
Lamin B1 Deficiency	Embryonic and adult mice	***Lmnb1* KO*^c^***			***Lmnb2* KO**		**Reciprocal knock-in *Lmnb1^B2/B2^***			Coffinier et al., 2010, 2011; Kim et al., 2011; Young et al., 2012; Lee et al., 2014b; Giacomini et al., 2016; Mahajani et al., 2017; Chen et al., 2019
		-Defective neuronal migration and survival, favored NSC differentiation to astrocytes, reduced axonal length -Smaller size of embryos, death shortly after birth, reduced cortical cellularity, thin cortical plate -NM*^d^* ruptures, cell death, lamin B2 overexpression does not prevent NM ruptures			-Bigger size of embryos, death shortly after birth, defective neural layering -Less frequent NM ruptures, lamin B2 overexpression abolishes NM ruptures		-Increased lamin B2 with less severe abnormalities than *lmnb1* KO			
*LMNB1* overexpression	Transfected embryonic mice NSC*^e^*	-Selective decrease of axonal growth-Increased neuron proportions								Mahajani et al., 2017

^*a*^NPC, neuronal progenitor cells.

^*b*^OPC, oligodendrocyte progenitor cells.

^*c*^KO, knock-out.

^*d*^NM, nuclear membrane.

^*e*^NSC, neural stem cells.

The next part of the review is dedicated to aging. Since the aim of this review is to give an overview of the role of lamin B1 from brain development to aging it is important to recap cellular senescence in the aging brain and then close with the role of lamin B1 in physiological and pathological aging.

## 4. Implications of cellular senescence in the aging brain

Aging is a physiological process of all living organisms that gradually deteriorates the overall health. Like any other organ, the brain displays signs of aging. One of the brain regions that has been extensively studied in the field of aging is the hippocampus. The reason behind this is that the hippocampus is involved in processes that are affected by aging, such as memory and cognition. Moreover, senescence in brain glial cells but also senescence-like states in post-mitotic neurons have been groundbreaking for understanding aging, cognitive decline, and neurodegeneration (Bettio et al., 2017; Sahu et al., 2022). In the following paragraphs, senescence in post-mitotic neurons and glial cells is described.

### 4.1. Senescence in post-mitotic neurons

Until recently, it was believed that neurons escape senescence because of the perception that senescence occurs only in dividing mitotic cells (Sahu et al., 2022). This belief was overturned after the discovery of senescence-like phenotype in terminally differentiated neurons (Jurk et al., 2012) that was characterized by markers of senescence such as senescence associated beta-galactosidase (SA-β-gal), monocyte chemoattractant protein-1 (MCP-1), gamma-H2A histone family member X (γ-H2AX), and 4-Hydroxynonenal (4-HNE) that led to the concept of amitosenescence (Wengerodt et al., 2019).

Long-term neuroglial senescent cultures showed upregulated SA-β-gal activity with senescence-associated secretory phenotype (SASP), sustained DNA damage in neuronal cells with high levels of reactive oxygen species (ROS), increased p21^*CIP*1^ expression, activated p38/mitogen activated protein kinases (MAPK) pathway, all of which are hallmarks of senescence (Jurk et al., 2012; Bigagli et al., 2015). Telomere length assessment of murine post-mitotic neurons also revealed cell-cycle dependent and cell-cycle independent telomere curtailment during brain aging. Although, telomere shortening is an important senescence aspect, it is still unknown whether this shortening is a senescence phenotype of the aging brain (Ain et al., 2018; Sah et al., 2021). Nonetheless, it has not been confirmed yet whether senescent post-mitotic cells are cleared from the brain or accumulate with age and act as drivers of age-related neurodegenerative diseases such as Alzheimer’s disease (AD) and Parkinson’s disease (PD) (Sahu et al., 2022).

### 4.2. Senescence in glial cells

Glial cells have been extensively studied in the field of brain cellular senescence. Starting with oligodendrocytes, it has been reported that oxidative stress and DNA damage can impair myelination by oligodendrocytes (Tse and Herrup, 2017). A study in aged rats displayed that recruitment and differentiation of OPC were compromised (Sim et al., 2002). Moreover, a recent study showed that extracellular vesicles of senescent astrocytes could not support oligodendrocyte maturation and differentiation *in vitro* (Willis et al., 2020). Therefore, it is evident that loss of myelin during aging can have adverse effects on glial and neuronal homeostasis (Sahu et al., 2022).

Regarding astrocytes, the term “astrosenescence” has been given (Cohen and Torres, 2019) to describe the role of astrocyte senescence in the aging brain. It has been reported that astrocytes are very sensitive to oxidative stress caused by hydrogen peroxide treatment and also proteasome inhibition induced senescent features such as SA-β-gal activation, p21, p16 and p53 elevated expression and several morphological alterations (Bitto et al., 2010). Furthermore, since astrocytes function closely with microglia for regulating synaptic maintenance and also for providing support to neurons, senescent microglia and astrocytes have exhibited upregulated p16^*INK*4*A*^ expression in recent studies of the hypothalamus of aged mice (Suda et al., 2021). Also, senescent microglia display SASP with a typical senescence-associated morphology (Marques et al., 2020) and enter the phase known as microglial dystrophy (Neumann et al., 2023).

## 5. Lamin B1 in the aging brain

As previously described, lamin B1 plays a crucial role in brain development. However, since aging is an inevitable process, it is important to distinguish the role of lamin B1 in physiological and pathological aging. In the following paragraphs, this topic will be elaborated to achieve an overview of the different roles of lamin B1 in the aging brain.

### 5.1. Lamin B1 decline during physiological brain aging

In the aging brain, the hippocampus has been most studied because of its involvement in memory and cognition which are properties affected by aging (Bettio et al., 2017; Sahu et al., 2022). Particularly, neurogenesis declines in the adult hippocampus with age resulting in cognitive and emotional impairments (Bedrosian et al., 2021). It has been reported that age-dependent downregulation of lamin B1 leads to age-related alterations in adult neural stem/progenitor cells (ANSPC) during hippocampal neurogenesis (Bedrosian et al., 2021). Intrinsic changes to ANSPC occurred as early as 5.5 months of age with losing lamin B1 at this point possibly being a driver of stem cell aging. Reduction of lamin B1 promoted the ANPC differentiation, reduced the return of ANSC into a quiescent state, and eventually depleted adult neurogenesis with lamin B1 influencing these transitions.

To investigate whether lamin B1 levels change in the context of adult hippocampal neurogenesis over the course of brain aging, immunohistochemical analyses on the mouse DG between 2 and 12 months of age were performed (Bedrosian et al., 2021). Compared to the levels of 2 months, lamin B1 levels in the SGZ were significantly reduced as early as 5.5 months of age even though ANSPC were still present in the SGZ. The early reduction in lamin B1 in ANSPC is correlated with early age-dependent reduction in adult hippocampal neurogenesis (Bergami et al., 2008). Thus, it was suggested that high levels of lamin B1 are important for adult hippocampal neurogenesis maintenance and age-dependent decline in lamin B1 contributes to age-related loss of hippocampal neurogenic capability.

In another study, declining lamin B1 with age was also observed, *in vitro* and *in vivo*, in mouse hippocampal NSC, whereas protein levels of SUN-domain containing protein 1 (SUN1) that has been previously implicated with Hutchinson-Gilford progeria syndrome, increased (around 4–6 months of age in rodents) (Bin Imtiaz et al., 2021). Importantly, changes in the expression of lamin B1 and SUN1 occurred simultaneously with around 80% decrease in neurogenesis from 2 to 8 months of age. Further, it was reported that lamin B1 and SUN1 regulate the strength of the endoplasmic reticulum diffusion barrier that acts as a mediator of asymmetric segregation of aging factors in proliferating NSC. Interestingly, it was suggested that recovering lamin B1 expression could be sufficient for rescuing proliferation deficits in aged NSC and for enhancing neurogenesis in the hippocampus of aged mice.

Regarding the cellular populations affected by loss of lamin B1, astrocyte senescence *in vitro*, in old mouse brains, and in post-mortem human brain tissue of elderly has been investigated (Matias et al., 2022). Severe reduction of lamin B1 in the dentate gyrus of aged mice, including hippocampal astrocytes (GFAP-positive), and in the granular cell layer of the hippocampus of post-mortem human tissue from non-demented elderly were reported. Lamin B1 reduction was associated with nuclear deformations, with increased incidence of invaginated nuclei and loss of nuclear circularity in senescent astrocytes *in vitro* and in the aging human hippocampus. Notably, differences in lamin B1 levels and astrocyte nuclear between the granular cell layer and polymorphic layer of elderly human hippocampus were identified, suggesting an intra-regional-dependent aging response of human astrocytes. Moreover, for further investigation of the effects of lamin B1 loss, astrocytes from mice were cultured for 30–35 DIV and tested for SASP markers such as β-gal, p16^*INK*4*a*^, matrix metalloproteinase-3 (MMP3), interleukin-6 (IL-6) and ROS and reactive nitrogen species (RNS). Increased expression of all the markers was observed, suggestive of lamin B1 associated senescence and increased inflammatory phenotype. Additionally, the first evidence on the impairment of neuritogenic and synaptogenic capacity of senescent astrocytes were reported. These findings were indicative of the involvement of senescent astrocytes in age-related synaptic decline.

In contrast to astrocytes, it was recently reported (Neumann et al., 2023) that aged microglia of the human brain (post-mortem tissues) with dystrophic morphology lack downregulation of lamin B1. Also, γ-H2AX, which is a widely used marker of cellular senescence, was not expressed in dystrophic microglia. Therefore, human aged microglia do not express conventional senescence markers associated with oxidative stress and their transformation to dystrophic cells is due to multiple pathogenic mechanisms. In Table 2, a summary of the effects of lamin B1 decline during physiological aging is depicted.

**TABLE 2:** Summary of the effects of lamin B1 decline in the different brain cells during physiological aging.

Lamin B1 decline during physiological brain aging				
**Cells**	**Hippocampal NSC*^a^***	**Hippocampal Astrocytes**		**Microglia**
**References**	Bedrosian et al., 2021; Bin Imtiaz et al., 2021	Matias et al., 2022		Neumann et al., 2023
**Models**	**Adult mice**	**Adult mice**	**Human post-mortem brain tissues**	**Human post-mortem brain tissues**
**Effects**	Reduced neurogenesis	-Increased invaginated nuclei -Increased inflammation and oxidative stress -Decreased neurites and synapses	Increased invaginated nuclei	-Lamin B1 reduction not observed -No dysmorphic morphology

^*a*^NSC, neural stem cells.

To sum up, it is evident that lamin B1 decrease in the aging hippocampus has severe implications not only in adult neurogenesis but also in astrocyte nuclear morphology and functions. Increasing lamin B1 levels seems to be a possible strategy for enhancing neurogenesis and regulating aging mechanisms but the exact increase needed without the onset of lamin B1 accumulation remains unknown. From the paragraphs related to brain development, the importance of maintaining balanced lamin B1 levels has been underlined since lamin B1 has been associated with age-related neurodegenerative diseases. These diseases, on one hand are characterized by overexpression of lamin B1 (Autosomal Dominant Leukodystrophy, ADLD) and on the other have decreased levels of lamin B1 (AD and PD). Thus, the following paragraphs are related to the role of lamin B1 in the pathological aging brain (Bedrosian et al., 2021; Bin Imtiaz et al., 2021; Evangelisti et al., 2022; Matias et al., 2022).

### 5.2. Lamin B1 and pathological brain aging

The growing range of human brain disorders linked to lamin B1 and the association of cellular senescence in the aging brain with the onset of neurodegeneration have recently piqued interest (Evangelisti et al., 2022; Sahu et al., 2022). The main adult-onset neurodegenerative disorder linked to lamin B1 is the rare disease known as ADLD (Padiath et al., 2006). Nevertheless, widely known age-related disorders, such as AD and PD, have also exhibited that lamin B1 mediates the disease progression (Frost et al., 2016; Chinta et al., 2018). The focus of the next paragraphs is on the role of lamin B1 in neurodegenerative age-related disorders such as ADLD, AD and PD.

Adult-onset ADLD is an age-related rare neurodegenerative disorder occurring at the fourth or fifth decade of life characterized clinically by early autonomic abnormalities, pyramidal and cerebellar dysfunction, and symmetrical progressive demyelination of the CNS with fatal outcome. ADLD is the main lamin-B linked disease and it is characterized by lamin B1 accumulation caused by tandem duplications or upstream deletions of the *LMNB1* gene (Padiath et al., 2006; Giorgio et al., 2015; Evangelisti et al., 2022; Neri et al., 2023). Overexpression of lamin B1 affects the inner nuclear membrane proteins, chromatin organization, and nuclear pore transport leading to abnormal nuclear morphology (Ding et al., 2021) and defective oligodendrocyte differentiation. Furthermore, duplications of *LMNB1* gene are related to the inhibition of myelin specific genes and to the activation of GFAP transcription (Dugas et al., 2006; Lin and Fu, 2009). Lamin B1 overexpression has also been related to a premature blockage in oligodendrocyte differentiation characterized by an alteration of the major myelin membrane lipoprotein PLP (proteolipid protein) (Lin and Fu, 2009).

For better understanding the role of overexpressed lamin B1 in demyelination, mouse models with ADLD features have been developed (Evangelisti et al., 2022; Neri et al., 2023). BAC transgenic model with two copies of murine wild-type lamin B1 (*Lmnb1^BAC^*) and transgenic mice overexpressing *Lmnb1* in different CNS lineages have been generated (Heng et al., 2013). It has been reported that overexpression of lamin B1 in oligodendrocytes and not in neurons or astrocytes was enough for the onset of ADLD associated deficits. Additionally, lamin B1 overexpression has been linked to motor deficits induction, aberrant myelin formation, axonal degradation, and demyelination associated with significant PLP1 decrease. Moreover, an oligodendrocyte-specific transgenic mouse overexpressing lamin B1 (Rolyan et al., 2015) displayed in oligodendrocytes a reduction of myelin-enriched lipids related to spinal cord myelin reduction. However, it has been demonstrated that transgenic mice do not display autonomic symptoms that are one of the main characteristics of ADLD onset. Thus, these models do not fully recapitulate the clinical phenotype of ADLD in humans (Lo Martire et al., 2018).

Studies on patient ADLD tissues have been insightful for understanding the pathobiological mechanisms of ADLD. Patient tissues (fibroblasts) have been studied for the implications of lamin B1 duplication in the whole-genome expression profile (Bartoletti-Stella et al., 2015). It was reported that *LMNB1* duplication affects transcription and alternative splicing of several genes including raver2 (RNA-binding protein), the dysregulation of which has been associated with abnormal development, reduced brain size and impaired corticogenesis in *Lmnb1*^Δ^ ^/Δ^ mice embryos (Vergnes et al., 2004; Coffinier et al., 2011; Kim et al., 2011; Evangelisti et al., 2022). Increased levels of lamin B1 and raver2 indicated ADLD as a “spliceopathy” with demyelination being the result of increased expression of the embryonic isoform of PLP1 during adulthood, which is important for maintaining myelin (Bartoletti-Stella et al., 2015). Moreover, a recent study displayed that the use of non-duplicated allele with allele-specific silencing by RNA interference (ASP-siRNA) may reduce the levels of *LMNB1* without excessively downregulating the gene (Giorgio et al., 2019). It was demonstrated that siRNA treatment in ADLD fibroblasts, murine oligodendrocytes overexpressing *LMNB1*, and reprogrammed neurons from patient fibroblasts effectively abrogated the ADLD-specific phenotype and therefore suggested a possible therapeutic strategy.

Although on the gene level a therapy has been suggested (Giorgio et al., 2019), the mechanisms behind ADLD remain unknown. Recent studies have contributed to the elucidation of the underlying disease mechanisms for targeted therapy toward specific cellular populations and biochemical pathways (Ratti et al., 2021a,b). The focus of these studies were astrocytes overexpressing lamin B1, for which it was shown that they contained several nuclear alterations absent from oligodendrocytes overexpressing lamin B1. The cells used for the experiments were MO3.13 (human oligodendrocytic cell line) and U87-MG (human glioblastoma astrocytic cell line) overexpressing *LMNB1*. The astrocytes were characterized by reduced secretion of leukemia inhibitory factor (LIF) that led to downregulation of pro-survival Janus kinase-signal transducer and activator of transcription protein 3 (Jak/Stat3) and phosphatidylinositol-3 phosphate kinase (PI3K)/Akt signaling pathways. Furthermore, studies on patient fibroblasts showed increased ROS production that could be additional to the ADLD pathogenesis. Moreover, it was demonstrated that lamin B1 increase leads to inactivation of glycogen synthase kinase (GSK)3β without β-catenin targets upregulation but with an *in vitro* reduction of astrocytic survival. Notably, astrocytes overexpressing lamin B1 displayed increased immunoreactivity for both GFAP and vimentin together with nuclear factor kappa-light-chain-enhancer of activated B cells (NF-κB) phosphorylation and c-Fos increase, suggesting astrocytes reactivity and substantial cellular activation. Thus, it is evident that astrocytes overexpressing lamin B1 could be playing a crucial role in ADLD progression (Evangelisti et al., 2022; Neri et al., 2023).

Regarding other age-related diseases, astrocyte dysfunction and unbalanced lamin B1 levels have also been observed in PD. Particularly, it has been shown that senescent astrocytes of PD patients were lamin B1 deficient, whereas neighboring cells (other than astrocytes) retained basal levels of lamin B1, suggesting that astrocytes could preferentially become senescent in PD brain tissue (Chinta et al., 2018). Moreover, reduction in lamin B protein levels and nuclear invaginations, also reported in physiologically aged astrocytes (Matias et al., 2022), have been shown in Tau-transgenic *Drosophila* brains and AD human brains and were associated with aberrant cytoskeleton-nucleoskeleton coupling and neuronal death (Frost et al., 2016). In neurons of AD patients, pathological tau leads to the stabilization of actin filaments, disrupting the linkers of the nucleoskeleton to the cytoskeleton complex and reducing lamin B. Consequently, this results in relaxation of constitutive heterochromatin and activation of cell-cycle in post-mitotic neurons leading to neuronal death (Frost et al., 2016; Evangelisti et al., 2022). Interestingly, even though PD and AD are not mainly caused by lamin B1 dysregulation (contrariwise to ADLD), lamin B1 loss could represent an early sign of age-associated brain pathology (Matias et al., 2022). A summary of the reported findings on lamin B1 in different age-related brain pathologies is depicted in the table below (Table 3).

**TABLE 3:** Summary of the effects of lamin B1 levels in different age-related brain pathologies.

Lamin B1 in pathological brain aging					
**Lamin B1**	**Decrease**		**Overexpression**		
**Disease**	**PD**	**AD**	**ADLD**		
**References**	Chinta et al., 2018	Frost et al., 2016	Heng et al., 2013; Rolyan et al., 2015; Lo Martire et al., 2018	Ratti et al., 2021a,b	Bartoletti-Stella et al., 2015
**Models**	**Human post-mortem brain tissue**	**Human post-mortem brain tissue and Tau-transgenic *Drosophila* brains**	**Transgenic mice**	**Human astrocytic, oligodendrocytic cell lines and** **patient fibroblasts**	**Patient fibroblasts**
**Effects**	Preferential astrocytic senescence	-Nuclear invaginations -Neuronal death	-Motor deficits -Dysfunctional lipid synthesis in oligodendrocytes (decreased PLP1*^a^*) -Autonomic symptoms not present (clinical aspect present in human ADLD patients)	-Nuclear alterations in astrocytes overexpressing lamin B1 not present in oligodendrocytes -Increased astrocytic immunoreactivity -Increased oxidative stress	-Increased raver2 -Altered splicing of PLP1

^*a*^PLP1, proteolipid protein 1.

## 6. Conclusions and future directions

Concluding, evidently lamin B1 is a crucial component of the developing and aging brain. Of note, B-type lamins are the only ones present in all brain cell types (neurons, astrocytes, oligodendrocytes, and microglia) throughout brain development (Takamori et al., 2007, 2018). With lamin B1 levels increasing during embryonic brain development (Takamori et al., 2007, 2018) and decreasing in the aged brain hindering adult neurogenesis and neuronal migration (Young et al., 2012; Bedrosian et al., 2021), it is conspicuous that lamin B1 levels should be finely tuned for physiological brain development (Mahajani et al., 2017). Moreover, in the aging brain lamin B1 decline has not only been associated with loss of hippocampal neurogenic capability (Bedrosian et al., 2021) and astrocytic nuclear anomalies (Matias et al., 2022) but also with neurodegenerative diseases such as AD (Frost et al., 2016) and PD (Chinta et al., 2018). However, overexpression of lamin B1 in the adult brain leads to the neurodegenerative disorder known as ADLD (Padiath et al., 2006). Therefore, it is important to maintain balanced levels of lamin B1 also in the adult brain to avoid the onset of neurodegeneration (Mahajani et al., 2017; Matias et al., 2022).

Future studies on balancing lamin B1 in the developing and aging brain will be insightful for ascertaining the onset of pathologies and for prospective therapeutic strategies with lamin B1 as the main target. It is important to highlight that most studies are mainly on *in vitro* and *in vivo* mouse models since human post-mortem brain materials are limited. Nevertheless, the level of uncertainty is increased due to the data extrapolation from rodent species (Stiles and Jernigan, 2010). For surpassing this limitation, the future of these studies includes the use of alternatives to animal models such as brain organoids. Brain organoids are promising new tools for studying molecular mechanisms and pharmaceutical developments in a three-dimensional (3D) environment (Bi et al., 2021). A recent study exhibited the use of dual-fluorescent reporter human induced pluripotent stem cells for the direct visualization of the transition status during neuron differentiation in 2D cultures and 3D brain organoids (Park et al., 2022). Moreover, 3D AD brain organoids have already been developed for not only studying the associated AD aging and neurodegeneration (Arber et al., 2021) but also for anti-AD drug evaluation (Yan et al., 2018). Consequently, such technologies could be applied for studying the effects of unbalanced lamin B1 not only on human cortical development but also on age-related neurodegenerative pathologies such as ADLD, AD and PD.

Finally, although lamin B1 is essential for physiological neural development and maturation (Mahajani et al., 2017), the importance of balancing lamin B1 levels in astrocytes should be highlighted. Astrocytes have increased levels of lamin B1 during brain development (Takamori et al., 2018) that during aging decrease, leading to aberrant nuclei and the onset of inflammation and oxidative stress (Matias et al., 2022). Interestingly, astrocytes overexpressing *LMNB1* as part of an ADLD model, were also characterized by abnormal nuclei and increased oxidative stress (Ratti et al., 2021b). Therefore, future studies on balancing the levels of lamin B1 in astrocytes will advance the knowledge regarding brain development and aging and more importantly provide new therapeutic strategies for neurodegenerative disorders such as ADLD and PD.

## Author contributions

F-DK: Conceptualization, Writing–original draft, Writing—review and editing. IN: Writing—review and editing. GR: Writing—review and editing. IR: Writing—review and editing. SM: Writing—review and editing. MS: Writing—review and editing. YK: Writing—review and editing. MM: Writing—review and editing. AF: Writing—review and editing. IC: Writing—review and editing. EG: Funding acquisition, Validation, Writing—review and editing. PC: Validation, Writing—review and editing. LM: Validation, Writing—review and editing. SR: Conceptualization, Funding acquisition, Validation, Writing–original draft, Writing—review and editing.
